# Supplementary figures and images for: Morphological and genetic diversity of maize landraces along an altitudinal gradient in the Southern Andes
Source: PLoS One. 2022 Dec 21;17(12):e0271424. doi: 10.1371/journal.pone.0271424 (PMC9770441; doi:10.1371/journal.pone.0271424)

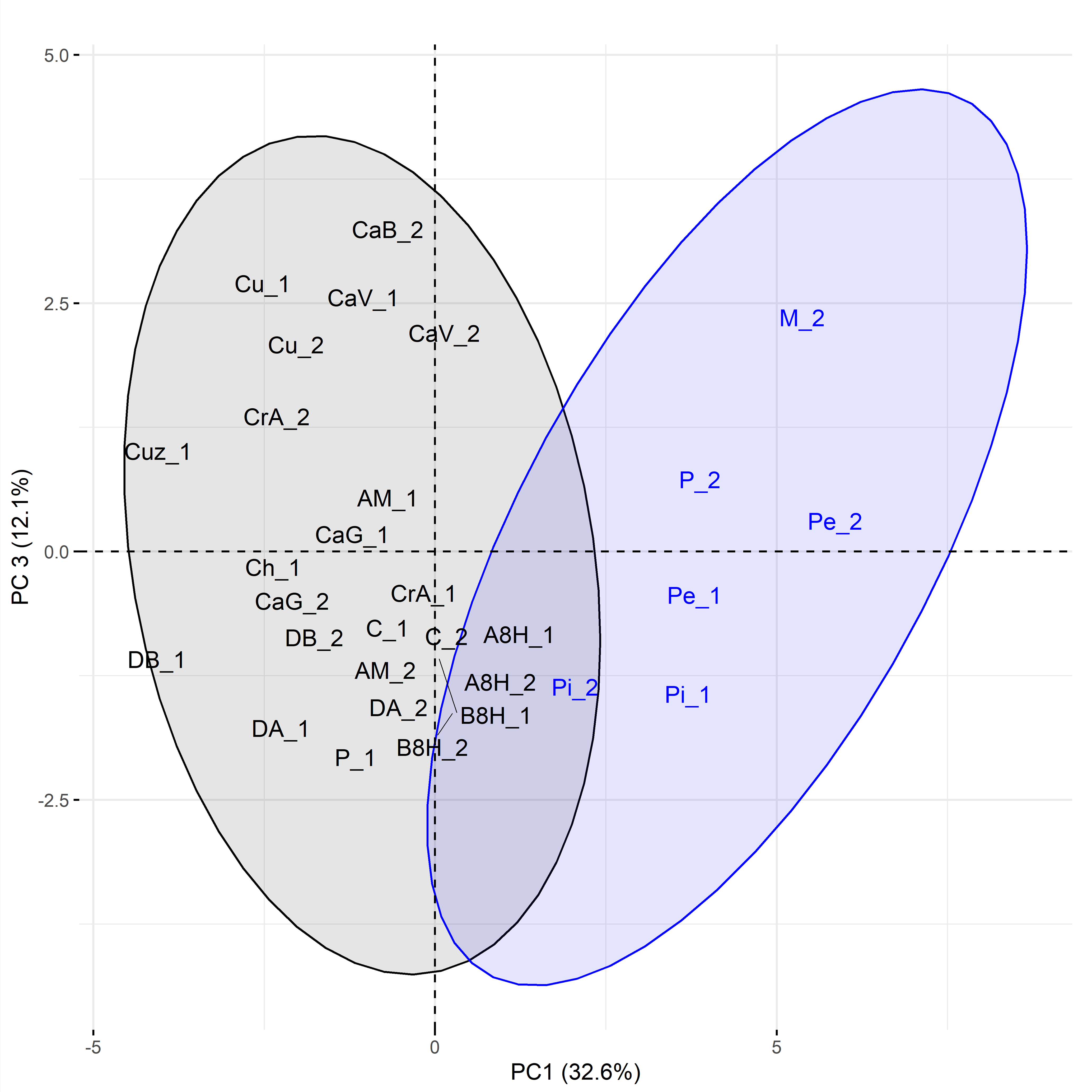

Supplement: S1 Fig — PC1 vs. PC3. (TIF) [file pone.0271424.s001.tif]

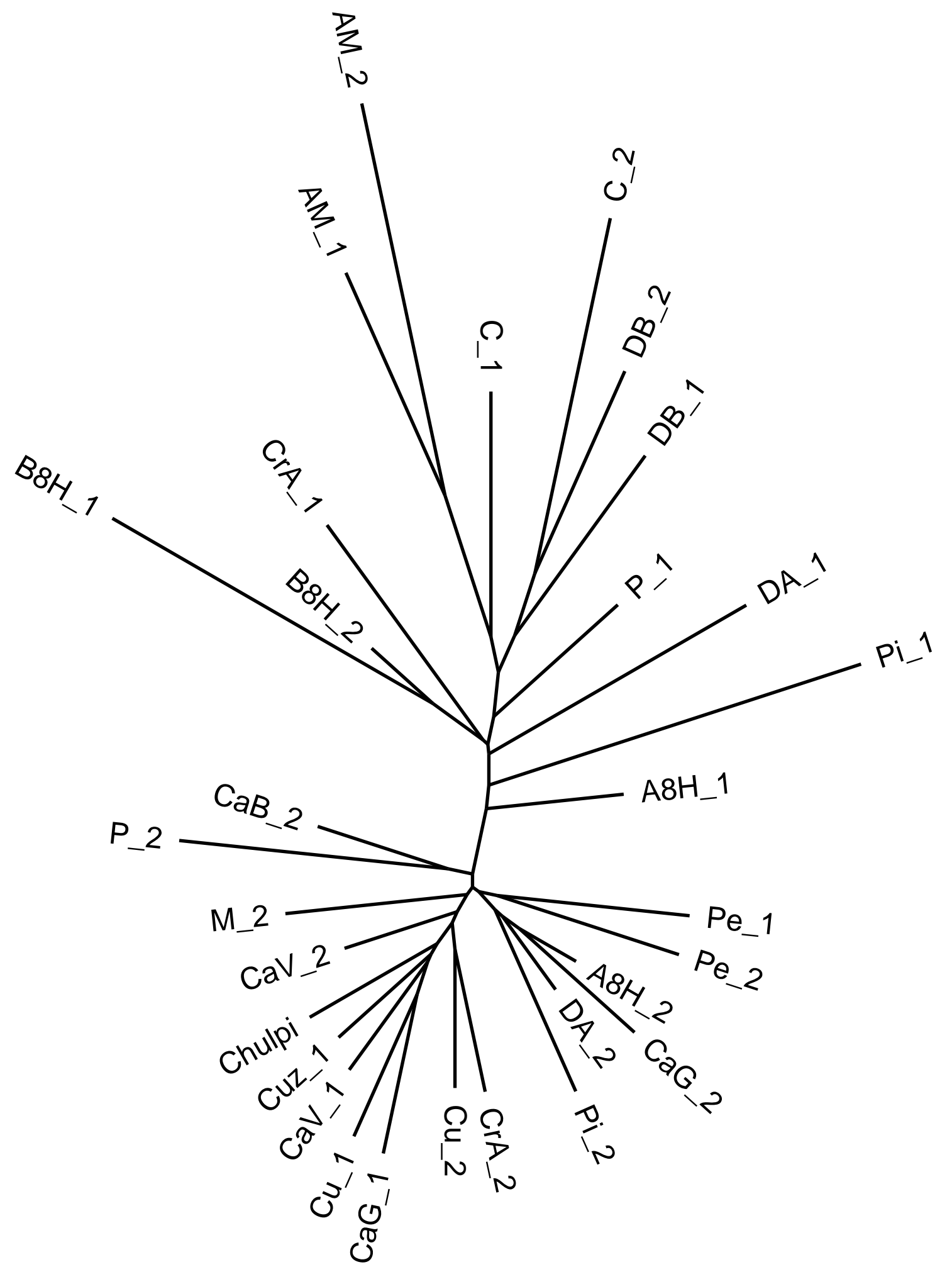

Supplement: S2 Fig — (PDF) [file pone.0271424.s002.pdf]
