# Supplementary material for: Morphological and genetic diversity of maize landraces along an altitudinal gradient in the Southern Andes
Source: PLoS One. 2022 Dec 21;17(12):e0271424. doi: 10.1371/journal.pone.0271424 (PMC9770441; doi:10.1371/journal.pone.0271424)
Supplement: S1 Table — (DOCX) [file pone.0271424.s003.docx]

| **Supplementary Table 1.** Voucher specimens, racial identification, endosperm type, collection sites, and altitude of the maize landrace accessions used for this study. | | | | | | | | | |
| --- | --- | --- | --- | --- | --- | --- | --- | --- | --- |
| **Accession** | **Landrace** | **ID** | **Endosperm type*** | **Collection site** | | | | **Altitude**  **(masl)** | **Number of individuals used for SSR analysis** |
|  |  |  |  |  |  | **Long** | **Lat** |  |  |
| 1 ARZM 09020 | Avati Moroti | AM_1 | floury | Yuto | Jujuy | -64.713127 | -23.506341 | 1500 | 14 |
| 2 ARZM 08025 | Avati Moroti | AM_2 | floury | El Ceibal | Salta | -64.85288 | -25.8881 | 1400 | 13 |
| 3 ARZM 08068 | Calchaqui | C_1 | flint | Buena Vista | Salta | -65.45399 | -25.56173 | 1120 | 8 |
| 4 ARZM 08116 | Calchaqui | C_2 | flint | Cafayate | Salta | -66.013094 | -26.061517 | 1990 | 14 |
| 5 ARZM 10082 | Perla | P_1 | flint | Amaicha del Valle | Tucumán | -65.95312 | -26.5974 | 1700 | 13 |
| 6 ARZM 09298 | Perla | P_2 | flint | Chijra | Jujuy | -65.36413 | -23.20968 | 2800 | 11 |
| 7 ARZM 09265 | Perlita | Pe_1 | semi-flint | Calete | Jujuy | -65.34289 | -23.239 | 2920 | 7 |
| 8 ARZM 09220 | Perlita | Pe_2 | flint | Malka | Jujuy | -65.40389 | -23.56848 | 2500 | 15 |
| 9 ARZM 10083 | Dentado Blanco | DB_1 | dent | Aristobulo del Valle | Tucumán | -65.95312 | -26.5974 | 1700 | 12 |
| 10 ARZM 09404 | Dentado Blanco | DB_2 | semi-dent | Tiraxi | Jujuy | -65.352473 | -24.004529 | 1760 | 14 |
| 11 ARZM 09178 | Amarillo de 8 Hileras | A8H_1 | floury | Barcena | Jujuy | -65.241832 | -65.241832 | 1800 | 14 |
| 12 ARZM 09213 | Amarillo de 8 Hileras | A8H_2 | floury | Quebrada de Juella | Jujuy | -65.42379 | -23.59162 | 2950 | 15 |
| 13 ARZM 09291 | Capia Blanco | CaB_2 | floury | Uquia | Jujuy | -65.36402 | -23.304633 | 2900 | 15 |
| 14 ARZM 09402 | Dentado Amarillo | DA_1 | dent | Tiraxi | Jujuy | -65.352473 | -24.004529 | 1760 | 15 |
| 15 ARZM 09381 | Dentado Amarillo | DA_2 | dent | El Volcan | Jujuy | -65.37050153 | -23.92936 | 2000 | 15 |
| 16 ARZM 09264 | Capia Variegado | CaV_1 | dent | Calete | Jujuy | -65.3297 | -23.2518 | 2920 | 12 |
| 17 ARZM 09212 | Capia Variegado | CaV_2 | dent | Juella | Jujuy | -65.42379 | -23.59162 | 2800 | 14 |
| 18 ARZM 10084 | Cristalino Amarillo | CrA_1 | flint | Amaicha del Valle | Tucumán | -65.92277 | -26.59075 | 1990 | 7 |
| 19 ARZM 09380 | Cristalino Amarillo | CrA_2 | flint | Tumbaya | Jujuy | -65.468881 | -23.857313 | 2094 | 15 |
| 20 ARZM 09179 | Blanco de 8 Hileras | B8H_1 | floury | Barcena | Jujuy | -65.241832 | -23.97141 | 1800 | 14 |
| 21 ARZM 09183 | Blanco de 8 Hileras | B8H_2 | flint | Barcena | Jujuy | -65.241832 | -23.97141 | 1800 | 17 |
| 22 ARZM 09257 | Morochito | M_2 | flint | Calete | Jujuy | -65.3297 | -23.2518 | 2920 | 13 |
| 23 ARZM 09206 | Capia Garrapata | CaG_1 | floury | Puerta de Juella | Jujuy | -65.41063 | -23.61469 | 2560 | 8 |
| 24 ARZM 09361 | Capia Garrapata | CaG_2 | flint | Maimará | Jujuy | -65.38132 | -23.57251 | 2375 | 16 |
| 25 ARZM 09277 | Pisingallo | Pi_1 | flint/pop | Calete | Jujuy | -65.3297 | -23.2518 | 2920 | 7 |
| 26 ARZM 09369 | Pisingallo | Pi_2 | flint/pop | Chicapa | Jujuy | -65.38658 | -23.53708 | 2410 | 14 |
| 27 ARZM 09209 | Cuzco | Cuz_1 | floury | Puerta de Juella | Jujuy | -65.41409 | -23.58519 | 2800 | 11 |
| 28 ARZM 09307 | Culli | Cu_1 | floury | Villa Perchel | Jujuy | -65.39257 | -23.57697 | 2580 | 10 |
| 29 ARZM 09197 | Culli | Cu_2 | flint | Tilcara | Jujuy | -65.3966 | -23.53422 | 2465 | 15 |
| 30 ARZM 09207 | Chulpi | Ch_1 | sweet | Puerta de Juella | Jujuy | -65.414879 | -23.584656 | 2560 | 11 |
| *Endosperm type was determined using CIMMYT IPGRI descriptors. masl: meters above sea level | | | | | | | | |  |
