# Supplementary material for: Morphological and genetic diversity of maize landraces along an altitudinal gradient in the Southern Andes
Source: PLoS One. 2022 Dec 21;17(12):e0271424. doi: 10.1371/journal.pone.0271424 (PMC9770441; doi:10.1371/journal.pone.0271424)
Supplement: S2 Table — (DOCX) [file pone.0271424.s004.docx]

| **Supplementary Table 2.** Characteristics of the SSR loci assayed for this study. Loci highlighted in gray were not included in the final analysis. | | | | |
| --- | --- | --- | --- | --- |
| **Locus** | **Repeat**  **Motif** | **Genomic Location**  **(bin)** | **Primers** | |
|  |  |  | ***Forward* 5´- 3´** | ***Reverse* 5´- 3´** |
|  |  |  |  |  |
| bnlg 149 | AG | 1 | CATCCTCCAAAAGCACTACGT | CAGCTGTCCGACACTTATTCTGTA |
| bnlg 1014 | AG | 1.01 | CACGCTTTTCAGACAGGAA | CGCCTGTGATTGCACTACAC |
| bnlg 1866 | AG | 1.03 | CCCAGCGCATGTCAACTCT | CCCCGGTAATTCAGTGGATA |
| bnlg 1017 | AG | 2.02 | ATTGGAGGATCTGCGTGAC | CAGCTGGTGGACTGCATCTA |
| bnlg 1018 | AG | 2.04 | CGAGGTTAGCACCGACAAAT | CGAGTAAATGCTCTGTGCCA |
| bnlg 1329 | AG | 2.08 | ATAGAATGGGATGTGGGCAA | TCCGATCATATCGGGAGATC |
| bnlg 1108 | AG | 3.08 | GGATTCCTTTATGACGGGGT | AGTAACAACCAAGGCATCGG |
| bnlg 1182 | AG | 3.09 | AGCCGAGTCAGTTCGAGGTA | CAGGGGCTTGAGGTGAGTTA |
| bnlg 1523 | AG | 3.02 | GAGCACAGCTAGGCAAAAGG | CTCGCACGCTCTCTCTTCTT |
| phi 072 | AAAC | 4 | ACCGTGCATGATTAATTTCTCCA GCCTT | GACAGCGCGCAAATGGATTGAACT |
| bnlg 252 | AG | 4.06 | CGTTCTCCGTACAGCACAGACCAACGT | CTCAGATGAACTCCTCAGCAGCTGTAGCCT |
| bnlg 1917 | AG | 4.1 | ACCGGAACAGACGAGCTCTA | TTTGCTTCCAACTCACATGC |
| bnlg 1287 | AG | 5.04 | GCCCTACCTGTTCTGTCTCG | TGTCCCATACCTCAACGTGA |
| bnlg 105 | AG | 5.02 | GACCGCCGGGACTGTAAGT | AGGAAAGAAGGTGACGCGTTTTC |
| bnlg 118 | CT | 5.07 | CTTCCAGCCGCAACCCTC | CCAACAACGCGGACGTGA |
| *bnlg 1732* | *AG* | *6.05* | *AACTTTTGGCATTGCACTGG* | *AACTTTTGGCATTGCACTGG* |
| bnlg 1740 | AG | 6.07 | TTTTCTCCTTGAGTTCGTTCG | ACAGGCAGAGCTCTCACACA |
| bnlg 2132 | AG | 7 | GGCGAGAGAGGCAAAGTTAA | GTCGCACAAGGGGATCAC |
| bnlg 1070 | AG | 7.03 | TTCCAGTAAGGGAGGTGCTG | TAAGCAACATATAGCCGGGC |
| bnlg 1194 | AG | 8.01 | GCGTTATTAAGGCAAGCTGC | ACGTGAAGCAGAGGATCCAT |
| phi 119 | AG | 8.02 | GGGCTCCAGTTTTCAGTCATTGG | ATCTTTCGTGCGGAGGAATGGTCA |
| bnlg 244 | AG | 9.02 | GATGCTACTACTGGTCTAGTCCAGA | CTCCTCCACTCATCAGCCTTGA |
| bnlg 1209 | AG | 9.05 | GTCCCGGGCAGAATAATACC | TTCCTCCTTGAAGTGCTCGT |
| bnlg 619 | CT | 9.07 | ACCCATCCCACTTTCCACCTCCTCCT | GCTTTCAGCGAATACTGAATAACGCGGA |
| bnlg 1526 | AG | 10.04 | ACGAGCGAGTGGAGAATAGG | AGCCCAGTACGTGGGGTC |
| bnlg 1360 | AG | 10.07 | TCTGCTCATCCACAACTTGC | AGAACGTGAAGGCTGAGCGTT |
| bnlg 1839 | AG | 10.07 | AGCAGACGGAGGAAACAAGA | TCTCCCTCTCCCTCTTGACA |
