# Supplementary material for: Morphological and genetic diversity of maize landraces along an altitudinal gradient in the Southern Andes
Source: PLoS One. 2022 Dec 21;17(12):e0271424. doi: 10.1371/journal.pone.0271424 (PMC9770441; doi:10.1371/journal.pone.0271424)
Supplement: S4 Table — (DOCX) [file pone.0271424.s006.docx]

| **Supplementary Table 4. Correlation coefficients between 19 quantitative traits in 30 maize landrace accessions from Northwestern Argentina.** Cells highlited in blue indicate significant values at the 5% level prior to Bonferroni corrections. Correlations that remained significant after Bonferroni corrections (p<0.0026) are highlighted in bold. | | | | | | | | | | | | | | | | | | | |
| --- | --- | --- | --- | --- | --- | --- | --- | --- | --- | --- | --- | --- | --- | --- | --- | --- | --- | --- | --- |
|  | PH | EH | NLA | TI | NL | ULL | ULW | VI | TL | TPL | TBS | NPBT | NSBT | NTBT | EPL | MNE | NRK | ED | NKR |
| PH | 1 |  |  |  |  |  |  |  |  |  |  |  |  |  |  |  |  |  |  |
| EH | **0.87** | 1 |  |  |  |  |  |  |  |  |  |  |  |  |  |  |  |  |  |
| NLA | **0.60** | 0.47 | 1 |  |  |  |  |  |  |  |  |  |  |  |  |  |  |  |  |
| TI | -0.18 | -0.24 | -0.05 | 1 |  |  |  |  |  |  |  |  |  |  |  |  |  |  |  |
| NL | **0.68** | **0.69** | **0.61** | 0.08 | 1 |  |  |  |  |  |  |  |  |  |  |  |  |  |  |
| ULL | 0.50 | 0.41 | 0.17 | 0.21 | 0.33 | 1 |  |  |  |  |  |  |  |  |  |  |  |  |  |
| ULW | 0.37 | 0.31 | 0.24 | 0.03 | 0.17 | **0.61** | 1 |  |  |  |  |  |  |  |  |  |  |  |  |
| VI | 0.12 | 0.07 | 0.21 | 0.02 | 0.16 | -0.13 | -0.42 | 1 |  |  |  |  |  |  |  |  |  |  |  |
| TL | **0.62** | 0.46 | **0.58** | -0.06 | 0.36 | **0.64** | 0.44 | -0.07 | 1 |  |  |  |  |  |  |  |  |  |  |
| TPL | -0.15 | -0.25 | -0.33 | -0.31 | -0.47 | -0.04 | 0.09 | -0.42 | 0.11 | 1 |  |  |  |  |  |  |  |  |  |
| TBS | **0.61** | **0.69** | **0.57** | -0.18 | 0.47 | 0.48 | 0.36 | -0.17 | **0.73** | 0.07 | 1 |  |  |  |  |  |  |  |  |
| NPBT | 0.41 | 0.51 | **0.60** | -0.09 | 0.43 | -0.11 | 0.04 | 0.14 | 0.37 | -0.17 | **0.60** | 1 |  |  |  |  |  |  |  |
| NSBT | 0.52 | **0.60** | 0.39 | -0.40 | 0.21 | 0.31 | 0.44 | -0.19 | **0.58** | 0.19 | **0.76** | 0.50 | 1 |  |  |  |  |  |  |
| NTBT | 0.27 | 0.23 | 0.11 | -0.22 | -0.04 | 0.20 | 0.10 | 0.07 | 0.29 | 0.25 | 0.31 | 0.09 | 0.38 | 1 |  |  |  |  |  |
| EPL | 0.26 | 0.11 | 0.28 | -0.01 | 0.02 | -0.02 | 0.26 | 0.06 | 0.25 | 0.15 | 0.13 | 0.26 | 0.31 | 0.09 | 1 |  |  |  |  |
| MNE | 0.10 | 0.10 | 0.07 | 0.13 | 0.08 | 0.28 | 0.51 | -0.39 | 0.26 | 0.09 | 0.25 | 0.28 | 0.39 | 0.13 | 0.37 | 1 |  |  |  |
| NRK | -0.04 | 0.18 | -0.31 | -0.24 | -0.41 | -0.06 | 0.09 | -0.23 | -0.13 | 0.27 | 0.18 | 0.06 | 0.39 | 0.33 | 0.08 | 0.24 | 1 |  |  |
| ED | **0.57** | **0.55** | 0.37 | -0.54 | 0.15 | 0.38 | 0.50 | -0.13 | 0.43 | 0.14 | **0.55** | 0.18 | 0.61 | 0.51 | 0.05 | 0.14 | 0.35 | 1 |  |
| NKR | 0.30 | 0.29 | 0.16 | -0.16 | 0.18 | 0.41 | 0.36 | 0.10 | 0.26 | 0.01 | 0.28 | 0.20 | 0.40 | 0.17 | 0.25 | 0.53 | 0.26 | 0.40 | 1 |
| **PH**: plant height, **NL**: number of leaves, **NLA:** number of leaves above the uppermost ear, **ULL**: uppermost leaf length, **ULW**: uppermost leaf width, **VI**: venation index, **TI**: tillering index, **TL**: tassel length, **TPL**: tassel peduncle length, **TBS**: tassel branching space, **NPBT**: number of primary branches on tassel, **NSBT**: number of secondary branches on tassel, **NTBT**: number of tertiary branches on tassel, **EH**: uppermost ear height, **EPL**: ear peduncle length, **ED**: ear diameter, **MNE**: mean number of ears , **NRK**: number of rows of kernels per ear, **NKR**: number of kernels per row | | | | | | | | | | | | | | | | | | | |
