# Supplementary material for: Morphological and genetic diversity of maize landraces along an altitudinal gradient in the Southern Andes
Source: PLoS One. 2022 Dec 21;17(12):e0271424. doi: 10.1371/journal.pone.0271424 (PMC9770441; doi:10.1371/journal.pone.0271424)
Supplement: S8 Table — (DOCX) [file pone.0271424.s010.docx]

| **Table S8**. Inference of the number of STRUCTURE clusters according to  Evanno’s Delta K (Evanno et al. 2005). | |
| --- | --- |
| K | Delta K |
| 1 | NA |
| **2** | **932.598018** |
| **3** | **41.628975** |
| 4 | 1.799392 |
| 5 | 0.394252 |
| 6 | 5.466209 |
| 7 | 0.101269 |
| 8 | 5.13228 |
| 9 | 0.373803 |
| 10 | NA |
